# Role of certain adipokines in the pathophysiology of hypertension

**DOI:** 10.3389/fphys.2026.1762472

**Published:** 2026-04-10

**Authors:** Ahmet Yilmaz, Basra Deniz Obay, Aysun Ekinci

**Affiliations:** 1 Department of Family Medicine, Faculty of Medicine, Dicle University, Diyarbakır, Türkiye; 2 Dicle University Medical Physiology Dicle Universitesi, Diyarbakır, Türkiye; 3 Dicle University Medical Biochemistry, Diyarbakır, Türkiye

**Keywords:** adipokine, biomarkers, cardioprotective effect, hypertension, inflammatory mediators

## Abstract

**Introduction:**

Adipose tissue is an active endocrine organ that secretes adipokines involved in metabolic regulation, inflammation, and cardiovascular homeostasis. Increasing evidence suggests that adipokine imbalance may contribute to the development of hypertension. This study aimed to compare circulating adipokine levels in newly diagnosed untreated hypertensive patients and normaotensive controls and to evaluate their association with blood pressure.

**Methods:**

This case–control study included 180 participants recruited from outpatient clinics at Dicle University Faculty of Medicine. The study population consisted of 96 newly diagnosed untreated hypertensive patients and 84 normotensive controls. Sociodemographic data and blood pressure measurements were recorded. Serum levels of leptin, intelectin, RBP-1, chemerin, visceral adipokine index, adiponectin, resistin, visfatin, and C-reactive protein were measured using ELISA kits. Statistical analyses included group comparisons, correlation analyses, ANCOVA adjusted for age, sex, and body mass index (BMI), and multivariate logistic regression.

**Results:**

Leptin, intelectin, RBP-1, resistin, and visfatin levels were significantly higher in hypertensive patients than in controls (p < 0.001). Adiponectin levels were slightly higher in hypertensive individuals, while chemerin, visceral adipokine index, and CRP showed no significant differences. Logistic regression analysis identified age, BMI, and resistin as independent predictors of hypertension.

**Conclusion:**

Several adipokines are elevated in hypertension, suggesting a potential role in blood pressure regulation. Among them, resistin appears to be an independent predictor of hypertension and may represent a potential biomarker for cardiovascular risk assessment.

## Introduction

Today, adipose tissue is recognized not merely as an energy depot, but as a hormonally active system involved in metabolic control (Coelho et al., 2013). The term “adipokine” was developed to describe a series of adipocyte-derived biologically active molecules capable of influencing tissue functions. These hormones play significant roles in various areas such as energy homeostasis, glucose and lipid metabolism, reproductive function, the cardiovascular system, and the immune system; they are directly related to nutritional status and have been shown to have direct effects on organ systems including the brain, liver, and skeletal muscle (Ouchi et al., 2011).

It is emphasized that hypertension is one of the most important preventable risk factors for cardiovascular diseases (CVD) and all-cause mortality worldwide. The increase in the prevalence of hypertension has been attributed to lifestyle factors such as population aging, unhealthy dietary habits, and lack of physical activity. Although obesity and adipose tissue dysfunction are recognized contributors to hypertension, the specific role of circulating adipokines in hypertension remains incompletely understood. Previous studies have reported associations between certain adipokines and blood pressure regulation; however, findings are inconsistent and based mostly on limited numbers of biomarkers or heterogeneous patient populations. In particular, the comparative evaluation of multiple adipokines in newly diagnosed and untreated hypertensive individuals remains limited in the literature (World Health Organization, 2023; GBD, 2019 Risk Factors Collaborators, 2020).

In patients with hypertension (HT), the levels of pro-inflammatory and anti-inflammatory adipokines are altered. The increase of adiponectin in HT may be a compensatory protective mechanism aimed at improving endothelial function, reducing oxidative stress, and increasing nitric oxide synthase. However, it remains unclear whether alterations in adipokine levels are independently associated with hypertension beyond established risk factors such as age, sex, and body mass index, or whether they primarily reflect underlying adiposity and metabolic status.

Through academic studies, adipokines have begun to be studied extensively in diseases. Nevertheless, the relationship between multiple adipokines and hypertension has not been fully clarified, and observational evidence examining these associations may help generate hypotheses regarding potential pathophysiological mechanisms. Furthermore, in this emerging world of adipokines, we believe that revealing the interaction with hypertension will form an infrastructure for future studies on the control and treatment of hypertension and associated diseases.

In this context, a case–control design provides an appropriate framework to examine differences in adipokine profiles between hypertensive and normotensive individuals and to evaluate their associations with hypertension status.

Our study aimed to determine adipokine levels from serum samples taken from hypertensive patients group and a healthy control group, and to compare serum adipokine levels between newly diagnosed untreated hypertensive patients and normotensive controls as the primary outcome, to reveal the effects of adipokines and their levels on the pathophysiology of hypertension, and to investigate their relationships with various measurable biological parameters. We further aimed to evaluate whether these adipokines are independently associated with hypertension after adjustment for age, gender, and body mass index. We hypothesized that specific adipokines would differ between hypertensive and normotensive individuals and would show significant associations with blood pressure measurements.

## Materials and methods

This study was conducted at Dicle University Faculty of Medicine Hospital. The population of this case-control study consisted of patients over the age of 18 applying to the Family Medicine and Cardiology outpatient clinics of Dicle University Faculty of Medicine Hospital.

Sampling for the sample size calculation, the “Sample Size Calculator Software” prepared by The Survey System was used, operating at a 95% confidence interval and a significance level of p ≤ 0.05. The minimum number of patients was determined to be 83 (https://www.surveysystem.com/sscalc.htm). We reached 96 patients, and additionally, 84 healthy volunteers were studied as the control group. A total of 180 individuals were included in the study. Inclusion criteria for the study were: the patient’s consent to participate, having the mental capacity to understand and make decisions, having a preliminary diagnosis of hypertension, not having a prior diagnosis of hypertension, not receiving any antihypertensive treatment, and not having diagnoses such as renal failure or heart failure.

Ethics Committee Approval for our study was obtained from the Dicle University Faculty of Medicine Non-Interventional Clinical Research Ethics Committee on 14.02.2019 with decision number 78.

Sociodemographic Data Form A prepared sociodemographic data form was used to query age, gender, marital status, height, weight, occupation, education level, medication use, smoking status, presence of diabetes or heart disease in the patient, and family history of hypertension, diabetes, or heart disease.

Measurement Method for the determination of blood pressure values in patients, after sufficient rest, the cuff was placed on the upper arm, 2 cm above the elbow. It was inflated manually by the researcher to approximately 200 mmHg, after which the valve was opened slowly to measure blood pressure. This process was repeated 3 times, and the average was recorded.

Laboratory Measurements *Serum Leptin, Visceral Adipokine, Intellectin, Chemerin, RBP-1, Adiponectin, Resistin, Visfatin, and C-reactive protein (CRP) Measurements:* Blood samples were collected in gel tubes. After collection, they were left at room temperature for 15 min to facilitate clotting. Blood samples were centrifuged at 5,000 rpm for 5 min. Serum was transferred to 1.5 ml polypropylene tubes and stored at −80 °C for further analysis.

Serum leptin, visceral adipokine, intellectin, chemerin, RBP-1, adiponectin, resistin, visfatin, and CRP levels were measured using YLA BIONT brand (Shanghai YL Biotech Co ELISA Kit LTD, China) commercial ELISA kits. Analyses were performed on a Biotek brand micro-ELISA device. To minimize assay variance, all measurements were performed on the same day. Tests were conducted according to the manufacturer’s instructions.

Statistics Statistical analysis applications were performed using the SPSS (Statistical Package for Social Sciences) for Windows 21.0 (IBM Corp., Armonk, United States) program. The conformity of data to normal distribution was checked using the Kolmogorov-Smirnov test. Descriptive statistical methods (Mean ± Standard deviation, median, count, percentage) were used when evaluating the data. Post hoc tests (for normally distributed data) were utilized to determine which parameters caused the difference in multiple groups. When comparing qualitative data, the Chi-square test was used. For examining relationships between data, the Pearson correlation test was used if the distribution was normal, and Spearman’s rho correlation test was used if the distribution was not normal. Results were within 95% confidence intervals, and differences between data were considered significant if p ≤ 0.05.

## Results

In the hypertensive patient, systolic and diastolic blood pressure medians were measured as 150 (110–190) mmHg and 90 (60–120) mmHg, respectively. In the control group, these values were determined to be 112.53 (90–140) mmHg and 72.56 (60–85) mmHg. The hypertensive patient was found to have significantly higher values in both blood pressure measurements compared to the control group (P < 0.001) (Table 1).

**TABLE 1:** Comparison of patient and control groups participating in the study in terms of blood pressure, adipokines and some values.

	Group	N	Median(min-max)	P
Sistolik tension	HPG	96	150(110–190)	
	Control	84	112,53(90–140)	P < 0.001
	Total	180		
Diastolik tension	HPG	96	90,00(60–120)	
	Control	84	72,56(60–85)	P < 0.001
	Total	180		
Leptin	HPG	96	7,84(1,13–980,88)	P < 0.001
	Control	84	2,40(1,15–46,92)	
	Total	180		
Visceral	HPG	96	1,01(0,23–5,14)	
	Control	84	0,79(0,1–2,65)	P = 0,41
	Total	180		
Intellectin	HPG	96	74,18(10,3–1438,78)	
	Control	84	37,53(10,1–131,54)	P < 0.001
	Total	180		
Chemerin	HPG	96	198,57(91,72–1380,13)	
	Control	84	177,57(131,46–852,25)	P = 0.347
	Total	180		
Rbp	HPG	96	109,64(44,97–5314,66)	
	Control	84	48,53(12,56–377,05)	P < 0.001
	Total	180		
Adiponektin	HPG	96	5,21(1,08–23,90)	
	Control	84	4,07(1,01–10,19)	P = 0.016
	Total	180		
Rezistin	HPG	96	529,32(119,97–3071,44)	
	Control	84	276,18(109,24–1078,04)	P < 0.001
	Total	180		
Visfatin	HPG	96	10,6134(1,25–93,92)	
	Control	84	5,77(1,77–27,04)	P < 0.001
	Total	180		
Crp	HPG	96	2,19(,04–6,56)	
	Control	84	2,19(,72–5,22)	P = 0.113
	Total	180		
BMI	HPG	96	28,72(18,43–41,62)	P < 0.001
	Control	84	25,13(16,22–47,38)	
	Total	180		

Hypertensive Patient Group, HPG, BMI, body mass index.

Levels of leptin, intellectin, RBP, resistin, and visfatin were shown to be significantly higher in the hypertensive patient with hypertension compared to the control group (e.g., leptin: 7.84 vs. 2.40; RBP-1: 109.64 vs. 48.53; P < 0.001 for all). Adiponectin levels were observed to be slightly higher in the hypertensive patient group compared to the control group (5.21 vs. 4.07; P = 0.016); however, no significant difference was detected between the two groups regarding chemerin and visceral adipokine levels (P = 0.347 and P = 0.41, respectively). No significant difference was revealed between groups in CRP levels either (P = 0.113). Additionally, the median BMI in the hypertensive patient group was determined to be higher than in the control group (28.72 vs. 25.13; P < 0.001).

According to blood pressure categories (normotensive—prehypertensive—hypertensive), there was a distinct increasing trend in leptin, RBP-1, resistin, and visfatin levels; the group differences for these were found to be statistically significant. Intellectin also showed an increase across blood pressure groups and was found to be significant (P = 0.02). Conversely, no significant difference was detected among blood pressure categories for visceral adipokine, chemerin, and CRP; statistical significance was not reached for adiponectin (p = 0.075). These findings indicate that the elevation of certain adipokine levels parallels blood pressure categories. Leptin, RBP-1, resistin, and visfatin levels increased from normotensive to prehypertensive and hypertensive; the changes in leptin and RBP-1 were particularly distinct and statistically significant (P < 0.001) (Table 2).

**TABLE 2:** Comparison of adipokine levels with blood pressure groups.

Variable (adipokine)	Normotensive	Pre hypertensive	Hypertensive	p
	Median(min-max)			
Leptin	2,60(1,15–65,5)	3,59(2,14–614,65)	7,4(1,13–980,88)	P < 0, 0001
Visceral	0,80(0,1–3,5)	0,91(0,42–5,06)	0,95(0,13–5,14)	P = 0.285
Intellectin	38,06( 10–336	41,49(10,3–956,49)	63,32(14,31–1438,78)	P = 0,02
Chemerin	180,60(111–852)p	175,74(98,86–1086,41)	189,55(91,72–1380,13)	P = 0.406
Rbp	49,2(12,56–810,48)	61,36(45,87–3744,76)	100,93(19,17–5314,66)	P < 0, 0001
Adiponektin	4,1(1,01–10,19)	4,78(1,08–21,12)	5,37(1,90–23,90)	P = 0.075
Rezistin	277,71(109,24–1078,04)	574,3(234,63–2682,53)	477,79(119,97–3071,44)	P < 0, 0001
Visfatin	5,95(1,77–29,63)	6,34(1,25–88,74)	9,1479(1,52–93,92)	P < 0, 0001
Crp	2,19(0,72–6,56)	2,19(0,04–5,49)	2,19 (05–6,24	P = 0.574

When normotensive and hypertensive groups were compared directly, median values for leptin, intellectin, RBP-1, resistin, and visfatin were all significantly higher in the hypertensive group (p < 0.0001 for all); this is a strong indicator of the relationship of these adipokines with hypertension (Table 3).

**TABLE 3:** Comparison of some adipokine levels in normal and high blood pressure groups.

Variable (adipokine)	Normotensive	Hypertensive	p
	Median(min-max)		
Leptin	2,60(1,15–65,59)	7,40 (1,13–980,88)	P < 00,001
Intellectin	38,06(10–33)	63,32(14,31–1438,78)	P < 00,001
Rbp	49,2(12,56–810,48)	100,93(19,17–5314,66)	P < 00,001
Rezistin	277,71(109,24–1078,04)	477,79(119,97–3071,44)	P < 00,001
Visfatin	5,95(1,77–29,63)	9,1479(1,52–93,92)	P < 00,001

Multivariate analysis of covariance (ANCOVA) was conducted to evaluate the differences in adipokine levels among hypertension groups (Normal, Pre-hypertensive, and Hypertensive) while controlling for the potential confounding effects of age, gender, and BMI. The analysis revealed that the majority of adipokines remained significantly associated with hypertension status even after adjustment (Table 4).

**TABLE 4:** Comparison of Adipokine Levels According to Hypertension Status (ANCOVA) The values below represent the Estimated Marginal Means, adjusted for the effects of age, body mass index (BMI), and gender.

Variable (adipokine)	Normal (n = 78) (Mean ± SE)	Pre-HT (n = 16) (Mean ± SE)	HT (n = 86) (Mean ± SE)	F value	p Value
Leptin (ng/mL)	−12.74 \ 23.16	93.20 \ 51.00	142.82 \ 24.80	9.936	<0.001
Visceral adiposity index (VAI)	0.93 \ 0.13	1.67 \ 0.28	1.51 \ 0.14	9.284	<0.001
Intellectin (ng/mL)	28.29 \ 33.84	184.28 \ 74.52	285.15 \ 36.24	12.987	<0.001
Chemerin (ng/mL)	221.43 \ 29.88	328.68 \ 65.80	326.38 \ 31.99	2.752	0.067
RBP1 (ng/mL)	−15.31 \ 95.19	461.84 \ 209.61	655.79 \ 101.93	9.566	<0.001
Adiponectin (μg/mL)	4.12 \ 0.48	4.85 \ 1.07	6.95 \ 0.52	7.935	0.001
Resistin (ng/mL)	275.34 \ 69.45	625.93 \ 152.94	925.58 \ 74.38	19.307	<0.001
Visfatin (ng/mL)	5.66 \ 2.19	15.92 \ 4.83	24.19 \ 2.35	16.929	<0.001

Means are evaluated at the following values for covariates: Age (47.09), BMI (27.09 kg/m^2), and Gender. HT: hypertension, SE: standard error.

A multivariate logistic regression model was constructed to identify the independent predictors of hypertension (HT) status (Normal vs. Hypertensive). The model was statistically significant (Omnibus test p < 0.001) and demonstrated a robust fit to the data (Hosmer-Lemeshow p = 0.278). Notably, the model achieved a high classification accuracy of 93.4%. The results indicate that age and Body Mass Index (BMI) are the strongest independent predictors of hypertension. Each one-unit increase in age was associated with a 1.15-fold increase in the risk of HT (15.6% increase), while each one-unit increase in BMI raised the risk by 1.20-fold (20.7% increase) (p < 0.05). Among the adipokines evaluated, Resistin emerged as a significant positive predictor of hypertension risk, even after adjusting for all demographic and anthropometric variables (age, gender, BMI) (p = 0.004). Every 1 ng/mL increase in Resistin levels was associated with a 1.01-fold increase in the likelihood of developing hypertension (OR: 1.010; 95% CI: 1.003–1.016). This finding suggests that Resistin may play a direct role in the pathogenesis of hypertension independent of obesity and age. While the Visceral Adiposity Index (VAI) and Chemerin were statistically significant in the model, their Odds Ratio (OR) values were below 1.0, which may suggest a protective orientation or a suppression effect within the multivariate model. Other markers, including Leptin, Visfatin, Adiponectin, and Intellectin, did not show significance as independent risk factors when evaluated alongside strong clinical predictors like age and BMI (p > 0.05) (Table 5).

**TABLE 5:** Logistic regression analysis of factors predicting hypertension risk (Adjusted Model) In this model, the independent effects of adipokines were analyzed by controlling for age, BMI, and gender.

Variable	B	Standard error (S.E.)	Wald	p-value	Odds ratio (exp(B))	95% confidence interval (CI)
Age	0.145	0.028	25.797	<0.001	1.156	1.093–1.222
BMI	0.188	0.087	4.724	0.030	1.207	1.019–1.431
Gender (Male)	−0.004	0.658	0.000	0.995	0.996	0.274–3.613
Visceral index (VAI)	−3.862	1.047	13.595	<0.001	0.021	0.003–0.164
Resistin	0.010	0.003	8.435	0.004	1.010	1.003–1.016
Chemerin	−0.020	0.010	4.173	0.041	0.980	0.961–0.999
Leptin	0.041	0.032	1.623	0.203	1.042	0.978–1.110
Visfatin	−0.081	0.106	0.581	0.446	0.922	0.748–1.136
Adiponectin	0.244	0.313	0.610	0.435	1.276	0.692–2.356
Intellectin	0.005	0.009	0.303	0.582	1.005	0.987–1.023
RBP1	0.002	0.003	0.436	0.509	1.002	0.996–1.009

Model Properties: Nagelkerke *R* ^2^ = 0.836; Hosmer and Lemeshow Test p = 0.278; Classification Accuracy = 93.4%.

Correlation analysis was performed for numerical measurement results, blood pressure values, adipokines, age, and body mass index (BMI) (Table 4). There is a strong positive correlation between systolic and diastolic blood pressure (r = 0.782, P < 0.01); additionally, age shows significant correlation with systolic (r = 0.634) and diastolic blood pressure (r = 0.523). BMI is also positively correlated with systolic and diastolic blood pressure (systolic r = 0.413; diastolic r = 0.368). Leptin shows very strong and significant positive correlations with intellectin (r = 0.741), RBP-1 (r = 0.863), resistin (r = 0.796), and visfatin (r = 0.824) (Table 6).

**TABLE 6:** Correlation analysis of measurement values.

Variables		Age	SBP	DBP	Leptin	Visceral	Intellectin	Chemerin	Rbp	Adiponektin	Rezistin	Visfatin	CRP	BMI
Age	r	1.000	0.634^**^	0.523^**^	0.324^**^	0.077	0.225^**^	-0.095	0.338^**^	0.110	0.292^**^	0.290^**^	-0.136	0.413^**^
	p		0.000	0.000	0.000	0.304	0.002	0.207	0.000	0.143	0.000	0.000	0.068	0.000
SBP	r	0.634^**^	1.000	0.782^**^	0.416^**^	0.068	0.239^**^	-0.073	0.418^**^	0.150^*^	0.390^**^	0.345^**^	0.041	0.390^**^
	p	0.000		0.000	0.000	0.365	0.001	0.332	0.000	0.044	0.000	0.000	0.584	0.000
DBP	r	0.523^**^	0.782^**^	1.000	0.376^**^	0.089	0.277^**^	-0.020	0.374^**^	0.156^*^	0.390^**^	0.360^**^	0.035	0.368^**^
	p	0.000	0.000		0.000	0.235	0.000	0.793	0.000	0.037	0.000	0.000	0.642	0.000
Leptin	r	0.324^**^	0.416^**^	0.376^**^	1.000	0.540^**^	0.741^**^	0.493^**^	0.863^**^	0.517^**^	0.796^**^	0.824^**^	0.076	0.208^**^
	p	0.000	0.000	0.000		0.000	0.000	0.000	0.000	0.000	0.000	0.000	0.309	0.005
Visceral	r	0.077	0.068	0.089	0.540^**^	1.000	0.589^**^	0.666^**^	0.653^**^	0.528^**^	0.607^**^	0.601^**^	0.039	0.005
	p	0.304	0.365	0.235	0.000		0.000	0.000	0.000	0.000	0.000	0.000	0.606	0.943
Intellectin	r	0.225^**^	0.239^**^	0.277^**^	0.741^**^	0.589^**^	1.000	0.665^**^	0.709^**^	0.633^**^	0.604^**^	0.685^**^	-0.018	0.092
	p	0.002	0.001	0.000	0.000	0.000		0.000	0.000	0.000	0.000	0.000	0.807	0.217
Chemerin	r	-0.095	-0.073	-0.020	0.493^**^	0.666^**^	0.665^**^	1.000	0.535^**^	0.625^**^	0.468^**^	0.484^**^	0.102	-0.066
	p	0.207	0.332	0.793	0.000	0.000	0.000		0.000	0.000	0.000	0.000	0.173	0.378
Rbp	r	0.338^**^	0.418^**^	0.374^**^	0.863^**^	0.653^**^	0.709^**^	0.535^**^	1.000	0.528^**^	0.794^**^	0.772^**^	0.048	0.190^*^
	p	0.000	0.000	0.000	0.000	0.000	0.000	0.000		0.000	0.000	0.000	0.521	0.011
Adiponektin	r	0.110	0.150^*^	0.156^*^	0.517^**^	0.528^**^	0.633^**^	0.625^**^	0.528^**^	1.000	0.433^**^	0.524^**^	0.040	0.044
	p	0.143	0.044	0.037	0.000	0.000	0.000	0.000	0.000		0.000	0.000	0.595	0.560
Rezistin	r	0.292^**^	0.390^**^	0.390^**^	0.796^**^	0.607^**^	0.604^**^	0.468^**^	0.794^**^	0.433^**^	1.000	0.771^**^	0.092	0.156^*^
	p	0.000	0.000	0.000	0.000	0.000	0.000	0.000	0.000	0.000		0.000	0.217	0.036
Visfatin	r	0.290^**^	0.345^**^	0.360^**^	0.824^**^	0.601^**^	0.685^**^	0.484^**^	0.772^**^	0.524^**^	0.771^**^	1.000	0.007	0.152^*^
	p	0.000	0.000	0.000	0.000	0.000	0.000	0.000	0.000	0.000	0.000		0.926	0.041
CRP	r	-0.136	0.041	0.035	0.076	0.039	-0.018	0.102	0.048	0.040	0.092	0.007	1.000	-0.062
	p	0.068	0.584	0.642	0.309	0.606	0.807	0.173	0.521	0.595	0.217	0.926		0.405
BMI	r	0.413^**^	0.390^**^	0.368^**^	0.208^**^	0.005	0.092	-0.066	0.190^*^	0.044	0.156^*^	0.152^*^	-0.062	1.000
	p	0.000	0.000	0.000	0.005	0.943	0.217	0.378	0.011	0.560	0.036	0.041	0.405	

Correlation is significant at the 0.05 level (2-tailed).

Correlation is significant at the 0.01 level (2-tailed).

SBP, systolic blood pressure; DBP, diastolic blood pressure.

## Discussion

The results suggest an association between the presence of hypertension and the elevation of specific adipokines. A distinct adipokine elevation was observed in the biochemical profile of hypertensive individuals. When normotensive and hypertensive patients were compared, leptin, intellectin, resistin, and visfatin were significantly higher in hypertensive. Resistin, visfatin, and intellectin showed positive relationships with each other; however, these findings are based on unadjusted group comparisons and bivariate correlations and therefore should be interpreted as exploratory associations rather than evidence of co-regulation or common pathophysiology. CRP showed weak or insignificant correlations with most variables; this suggests that CRP, a general inflammation marker, does not move directly in parallel with adipokines and blood pressure in this context. Correlations indicate that certain adipokines, particularly leptin, were positively associated with both other adipokines and with blood pressure and BMI; this may reflect shared metabolic or inflammatory processes rather than independent causal effects. Given the observational case-control design and the absence of multivariable adjustment for potential confounders such as age, BMI, and treatment status, causal or mechanistic inferences cannot be made.

It has been proven that leptin participates in autonomic nervous system control partly by increasing renal sympathetic nerve activity (RSNA). It is thought that leptin resistance exists only in satiety and weight gain mechanisms, while there is no resistance in mechanisms causing sympathetic activation and hypertension (Simonds et al., 2014). In the kidney, leptin affects blood pressure via two different pathways: the renal sympathetic activation pathway and the nitric oxide pathway (Rahmouni, 2016; Mark et al., 2018). These effects vary according to the duration of hormone exposure. Acute exposure does not increase blood pressure, but blood pressure increases with prolonged exposure. It is thought that sympathetic activation increases sodium retention, thereby predisposing to hypertension. At the same time, sympathetic activation exerts a pressor effect. However, it has also been observed that leptin stimulates NO release, which acts counter to these mechanisms (Frühbeck et al., 2016; Beltowski, 2016). Vecchione et al. (Mark et al., 2018) showed that leptin stimulates NO production in endothelial cells and blood vessels. In chronic exposure, both RSNA and blood pressure increase. Chronic hyperleptinemia reduces natriuresis and urinary excretion of NO metabolites. Long-term hyperleptinemic states such as obesity increase systemic and intrarenal oxidative stress levels and lead to NO deficiency (Hall et al., 2015). It chronically increases sodium retention (Seravalle and Grassi, 2017). These mechanisms reported in previous studies provide biological context; however, the present study does not directly evaluate these pathways.

In our study, the mean serum leptin level was 7.84 in the hypertensive patient group and 2.40 in the control group and this difference was found to be statistically significant. When patients were clustered according to blood pressure, the mean leptin level was 2.60 in normotensives, 3.59 in prehypertensives, and 7.4 in hypertensives. Consistent with previous reports, leptin levels were higher in hypertensive individuals; however, this finding represents an association and does not establish a causal role in the development of hypertension. Additionally, in the correlation analysis, a positive correlation was seen between both systolic and diastolic blood pressure levels and leptin levels. This indicates that high leptin levels in obese individuals may be associated with higher blood pressure levels. However, these relationships were not adjusted for potential confounders and should therefore be interpreted cautiously. Consequently, as the severity of obesity increases, the stage of hypertension may also be associated, but no causal inference can be drawn from the present data. In a study examining the effect of systolic and diastolic blood pressure on cardiovascular mortality, it was seen that blood pressure levels in the prehypertensive range increased cardiovascular events and stroke (SPRINT Research Group et al., 2015).

Omentin (intelectin) is released from epicardial periadventitial adipose tissue and protects against atherosclerosis by activating the eNOS system (Greulich et al., 2013). Studies have shown associations between lower omentin levels and cardiometabolic risk including hypertension and endothelial dysfunction (Saely et al., 2015; Zhong et al., 2017). Omentin induces endothelium-dependent vasodilation via nitric oxide pathways (Yamawaki et al., 2010). This indicates that omentin levels have been suggested to be involved in the pathogenesis of hypertension (Greulich et al., 2013). In our study, mean omentin (intelectin) levels were 74.18 in the patient group, while they were 37.53 in the control group. It was found to be 2.60 in normotensives, 3.59 in prehypertensives, and 7.4 in hypertensives. This difference was statistically significant. This situation does not align with the literature information. In the correlation analysis, intellectin was found to be correlated with age, systolic and diastolic blood pressures, and other adipokines except CRP. It was not found to be correlated with BMI. These findings should be interpreted cautiously, as the study design does not allow clarification of the direction or mechanism of these associations. Omentin levels have been shown to be inversely associated with metabolic risk factors (Shibata et al., 2012). Omentin is released mostly from visceral adipose tissue, but the expression of the gene synthesizing omentin decreases in obesity. This increases insulin resistance.

Serum resistin concentration is higher in patients with Diabetes Mellitus compared to normal individuals and those with prediabetes (Zhang et al., 2017). Studies on serum resistin levels have shown that it is present in low concentrations in adipose tissue and is not associated with parameters such as body weight and BMI (Verma et al., 2003; Reilly et al., 2005; Jamaluddin et al., 2012; Muse et al., 2015). Resistin inhibits insulin-stimulated glucose uptake in adipose tissue. Anti-resistin antibodies increase this. Therefore, it is thought that resistin may increase insulin resistance (Verma et al., 2003). In our study, mean serum resistin levels were found to be 529.32 in the hypertensive patient group and 276.18 in the control group, and this difference was statistically significant. It was found to be 277.71 in normotensives, 574.3 in prehypertensives, and 477.79 in hypertensives. This difference was also found to be statistically significant. However, interestingly, resistin did not show an increase consistent with the hypertension stage like other adipokines. It reached the highest level in the prehypertensive group. Resistin, like others, was found to be correlated with age, blood pressure, and other adipokines except CRP. It was not found to be correlated with BMI. These results indicate an association but do not support a consistent stage-dependent relationship with hypertension.

Chemerin plays a pro-inflammatory role and shows a positive correlation with pro-inflammatory cytokines IL-6, CRP, and TNF-α. Chemerin contributes to the inflammation process by activating macrophages and inflammatory cytokines (Lehrke et al., 2009). It is suggested that chemerin causes a local paracrine inflammatory response in obesity. By causing the release of pro-inflammatory and pro-diabetic adipokines, chemerin disrupts adipose tissue metabolism and causes negative systemic effects (Landgraf et al., 2012). Just as chemerin increases the inflammatory response in adipose tissue, it also increases the inflammatory response of macrophages within tumors (Bozaoglu et al., 2007). There are very few studies conducted on chemerin and hypertension. The plasma level of chemerin is strongly associated with blood pressure in normal individuals and those with metabolic syndrome, but whether it is an independent risk factor for hypertension is not certain. Chemerin levels have also been found to be high in renal failure (Landgraf et al., 2012). In our study, the mean chemerin level was 198.57 in the hypertensive patient group, while it was 177.57 in the control group. No statistically significant difference was found. Consistent with these results, no independent association between chemerin levels and hypertension was demonstrated in our study. In the correlation analysis, chemerin was not found to be correlated with either systolic or diastolic blood pressures. While correlated with other adipokines except CRP, no correlation was found with parameters such as age and BMI. In our study, no relationship was found between chemerin levels and hypertension. In the study by Ademoğlu et al., chemerin was found to be correlated with BMI and TG (Bozaoglu et al., 2007). Kort et al. also found a positive correlation between chemerin, BMI, and HOMA-IR (Neves et al., 2015). However, in our study, chemerin did not show a correlation with BMI either.

Apelin is an adipokine involved in cardiovascular regulation and has hypotensive and vasodilatory effects mediated through endothelial nitric oxide pathways (Tatemoto et al., 1998; Kuba et al., 2007). It is thought that Apelin acts in a paracrine manner; it has effects on water intake and the hypothalamic-pituitary axis. Additionally, it is thought to have hypotensive effects in the cardiovascular system, effects that increase myocardial contraction, and effects on angiogenesis. Apart from this, it is associated with obesity and insulin resistance (Tatemoto et al., 1998).

Visfatin (NAMPT) has been linked with vascular inflammation, endothelial dysfunction, and hypertension in recent observational and mechanistic studies (Fukuhara et al., 2005; Garten et al., 2015; Montecucco et al., 2013; Dahl et al., 2012). In our study, mean visfatin levels were found to be 10.6134 in the hypertensive patient group, while they were 5.77 in the control group, and this difference was found to be statistically significant. The visfatin level was found to be 5.95 in normotensives, 6.34 in prehypertensives, and 9.1479 in hypertensives. Additionally, visfatin levels were found to be correlated with age, blood pressure, and other adipokines except CRP, but not correlated with BMI. These findings support an association between visfatin levels and hypertension status, although the observational design prevents conclusions regarding causality or underlying mechanisms.

Overall, the present study provides descriptive and exploratory evidence of associations between adipokines and hypertension. The findings should be interpreted in light of the study’s methodological limitations, including the observational case-control design, the use of unadjusted group comparisons and bivariate correlations, and the lack of multivariable analyses controlling for potential confounders. Future studies using longitudinal designs and multivariable modelling are required to clarify the independent and causal roles of adipokines in hypertension.

## Conclusion

In conclusion, all adipokines except CRP were found to be correlated with each other. The adipokines showing correlation with both systolic and diastolic blood pressure are the same; these are leptin, intellectin (omentin), RBP-1, resistin, and visfatin. Among all adipokines, only leptin was found to be correlated with BMI. The adipokines showing correlation with age are leptin, intellectin, RBP-1, resistin, and visfatin, which is the same group correlated with blood pressure.

Certain adipokines—particularly Resistin, Visfatin, and Intellectin—serve as significant biomarkers of hypertension. Their association remains robust after controlling for age and obesity, highlighting their potential role in the independent pathophysiology of hypertensive disease.

This study demonstrates that beyond the conventional risk factors of age and body weight, elevated Resistin levels serve as an independent biochemical marker of hypertension risk. The Nagelkerke *R* ^2^ value of 0.836 indicates that the model explains a substantial 83.6% of the variance in hypertension status.

We foresee that in the development processes of hypertension and during diagnostic processes in outpatient clinics, resistin levels could be applied among routine tests, and related additional studies focusing on this subject can be conducted.

This study was produced from the thesis titled “THE ROLE OF CERTAIN ADIPOKINES IN THE PATHOPHYSIOLOGY OF HYPERTENSION”. Financial support was provided by the Dicle University Faculty of Medicine Scientific Research and Projects Coordination Unit (DÜBAP) under project number Tıp.19.013 during the conduct of the thesis.

## Data Availability

The original contributions presented in the study are included in the article/supplementary material, further inquiries can be directed to the corresponding author.
